# An online tiered screening procedure to identify mental health problems among refugees

**DOI:** 10.1186/s12888-022-04481-2

**Published:** 2023-01-03

**Authors:** Jennifer Meurling, Elisabet Rondung, Anna Leiler, Elisabet Wasteson, Gerhard Andersson, Derek Richards, Shervin Shahnavaz, Anna Bjärtå

**Affiliations:** 1 Department of Psychology and Social work, Mid Sweden University, 831 25 Östersund, Sweden; 2 Department of Behavioral Sciences and Learning, Linköping University, 581 83 Linköping, Sweden; 3 School of Psychology, Trinity College Dublin, College Green, Dublin 2, Ireland; 4 Centre for Psychiatry Research, Department of Clinical Neuroscience, Karolinska Insititutet, & Stockholm Health Care Services, Region Stockholm, 171 77 Stockholm, Sweden

**Keywords:** Digital mental health, Tiered screening, Online assessment, Refugees

## Abstract

**Background:**

Many refugees suffer from mental health problems due to stressful and traumatic events before, during, and after migration. However, refugees are facing a wide variety of barriers, limiting their access to mental health care. Internet-based tools, available in several languages, could be one way to increase the availability of mental health services for refugees. The present study aimed to develop and test a screening tool to screen for clinically relevant symptoms of psychiatric disorders common among refugees (i.e. Depression, Anxiety, Post-traumatic stress disorder, and Insomnia). We, designed, translated, and adapted an internet-based tiered screening procedure suitable for use with the largest refugee populations residing in Sweden. The tool aims to accurately identify symptoms of mental distress (Tier 1), differentiate between symptoms of specific psychiatric disorders (Tier 2), and assess symptom severity (Tier 3). We tested the overall efficiency of using a tiered screening procedure.

**Methods:**

Seven hundred fifty-seven refugees residing in Sweden, speaking any of the languages Arabic, Dari, Farsi, English, or Swedish, completed an online questionnaire following a three-tiered procedure with screening instruments for each tier. In this study, the Tier 3 scales were used as reference standards for clinically relevant symptoms, to evaluate screening efficiency in terms of accuracy and reduction of item burden in previous tiers.

**Results:**

The results show that the tiered procedure could reduce the item burden while maintaining high accuracy, with up to 86% correctly assessed symptoms and few false negatives with moderate symptoms and above (at most 9%), and very few with severe symptoms (at most 1.3%).

**Discussion:**

This study generated an accurate screening tool that efficiently identifies clinically relevant symptoms of common psychiatric disorders among refugees. Using an adapted online tiered procedure to screen for multiple mental health issues among refugees has the potential to facilitate screening and increase access to mental health services for refugees. We discuss the utility of the screening tool and the necessity of further evaluation.

## Background

Worldwide, people are forcibly displaced from their homes due to persecution, conflict, violence, human rights violations, or events alarming public order. Forcibly displaced persons who have crossed the national border and need international protection are generally referred to as refugees [1]. For the past decade, there has been a dramatic increase in refugees, with numbers that keep reaching record highs each year [2]. The year 2022 does not seem to be an exception to this. With the recent developments in Europe, another 7.8 million people who have fled the war in Ukraine [3] can be added to the list. Most refugees experience extreme stress and have also experienced one or more traumatic events before or during the flight [4]. However, the mental health of refugees is also affected by post-migratory factors, such as long asylum processes and temporary residence permits, and by resettlement stressors, such as unemployment or social isolation [5, 6].

Previous research has shown a coherent picture of elevated mental health problems among refugees worldwide [7, 8]. For example, refugees are at 10 times higher risk of developing Post-traumatic stress disorder (PTSD) than the general population. Also, the prevalence of PTSD, Depression, and Anxiety among adult, child, and adolescent refugees, is substantially higher compared to the general population in high-income countries [9–11]. Experiencing trauma has both immediate and long-term effects on sleep [12], and sleep disturbances, including Insomnia, are common among refugees [13–15]. Since sleep disorders are bidirectionally related to other mental health symptoms [16] and are often reported by refugees when asked about problematic aspects of their situation [17], assessing them is crucial. Refugees residing in Sweden show similar patterns of mental health problems as refugees worldwide. Symptoms of PTSD, Anxiety, Depression, general psychological distress [18, 19] and bad sleep quality [20] reach similar levels as in international studies.

Additionally, refugees frequently report comorbid psychiatric symptoms [8, 18, 19, 21], further increasing individual suffering and functional impairment, and psychiatric symptoms and disorders persist over time [22]. There is thus a pressing need for evidence-based, efficient, and feasible methods for identifying, assessing, and treating mental health problems among refugees regardless of time displaced.

Although many refugees urgently need mental health interventions, refugees residing in European countries show an under-utilisation of mental health services [23, 24]. This treatment gap between service provision and service access is affected by multiple barriers. Foremost among the reported barriers are language and communication difficulties, including the use of an interpreter [25, 26]. Refugees also report other substantial barriers to seeking and receiving mental health care, such as fear of stigma and issues relating to transportation, financial challenges and difficulties locating services [27].

Internet-based tools for refugee mental health are an emerging and promising field. With the potential of increasing accessibility and convenience, internet-based tools offer a possibility to increase refugees’ access to and use of mental health services. Tools for clinical assessment, including self-report questionnaires, have proved helpful in identifying psychological symptoms, as well as for diagnostic screening and referral [28, 29]. However, internet-based assessments adapted for refugee populations are limited, despite emerging evidence supporting the use of digital tools for this group. Digital screening procedures have been found highly acceptable, less time-consuming, and psychometrically comparable to paper-and-pencil questionnaires [30, 31].

Another advantage of the online format is the possibility to use an adaptive and automated tiered model, with an initial brief general screener followed by longer, more specific screening instruments only for individuals that meet specified criteria in the previous step. As suggested by Batterham et al. [32], such a format renders a possibility to screen for several psychiatric disorders simultaneously while potentially reducing the item burden.

The present study aimed to develop and evaluate the validity of an online tiered screening procedure for mental health problems, adapted for refugees. The screening procedure identifies symptoms of mental distress in the first tier, differentiates between symptoms of psychiatric disorders in the second tier, and indicates the severity of symptoms in the third tier. In this study, the results from the third tier are used as reference standard for clinically relevant symptoms of Depression, Anxiety, PTSD and Insomnia. The study tested the screening efficiency of the tiers using two different models, and screening efficiency was evaluated through screening accuracy and item burden reduction.

## Methods

This study is part of a large project aiming to develop and evaluate online psychological assessment and intervention methods for young adults, including adolescents from 15 years of age, with refugee backgrounds. As part of that project, we conducted a cross-sectional study with refugees in Sweden. Between May and September 2020, we collected participant data via an online questionnaire in languages corresponding to the largest refugee groups residing in Sweden (i.e. Arabic, Dari, Farsi), and Swedish and English [33]. Data were collected and stored in secure servers at Linköping University. The study has been reviewed and approved by the Swedish Ethical Review Authority (2020–00214).

### Recruitment and participants

We recruited individuals aged 15 years or above, who answered yes to a question asking them whether they had fled their home countries due to war, conflict, persecution, threat or similar reasons. That is, refugee status was self-defined by answering this question. We translated study materials into Arabic, Dari, Farsi, and English, and we used several sources for recruitment (e. g. social media, asylum housings, schools, and other meeting points for refugees). Because the survey was anonymous, no support could be offered directly to distressed individuals. However, we provided contact details to a healthcare professional, who could offer personalised guidance on how to seek care. A convenience sample of 823 respondents from all 21 regions of Sweden completed the questionnaire. We excluded data from 66 participants, of which 64 indicated that they did not have a refugee background, and two were under 15 years of age. The remaining 757 had a mean age of 32 (*SD* = 11, Range 15–72). Respondents had a variety of nationalities, with a majority from Syria and Afghanistan (see Table 1 for sample characteristics), and 51.9% answered the questionnaire in Arabic, 17.8% in Swedish, 12.5% in Dari, 9.9% in Farsi, and 7.8% in English.

**Table 1:** *Sample Characteristics*

	Frequency	Percent
***Nationality***		
Syria	265	35.0
Afghanistan	187	24.7
Palestine	44	5.8
Iran	36	4.8
Iraq	36	4.8
Eritrea	31	4.1
Somalia	19	2.5
Yemen	19	2.5
Sudan	10	1.3
Kurdistan	9	1.2
Ethiopia	8	1.0
Stateless	32	4.2
*N* < 5*	61	8.1
***Gender***		
Male	471	62.2
Female	283	37.4
Other	3	0.4
***Marital Status***		
Single	348	46.0
Married/Partner	313	41.3
Divorced/Separated	67	8.9
Widowed	3	0.4
Other	26	3.4
***Education***		
High school	255	33.5
University MA	198	26.2
University BA	144	19.0
Primary school	106	14.0
Vocational training	42	5.5
Other	12	1.6
***Residence permit (RP)***		
Permanent	378	49.9
Temporary	206	27.2
No RP	173	22.9

*Albania, Algeria, Bosnia, Burkina Faso, Burundi, Cameroon, Chile, Congo, Egypt, El Salvador, Georgia, Indonesia, Kazakhstan, Kenya, Kuwait, Lebanon, Libya, Morocco, Nigeria, Pakistan, Rwanda, Sierra Leone, Tunisia, Turkey, Uganda, Ukraine, United Arab Emirates, Uruguay, Uzbekistan

### Materials

#### The i-TAP

**Fig. 1:**
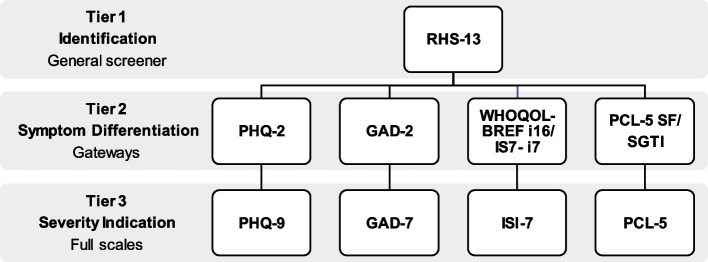
Illustration of the tiered design of the i-TAP, with the symptom scales used in each tier

The internet-based tiered assessment procedure, herein called i-TAP, has been developed as part of a project focusing on digital mental health interventions for refugees. The purpose was to target clinically relevant symptoms of Depression, Anxiety, PTSD, and Insomnia among refugees, using an online tiered screening procedure. This procedure has three tiers (see Fig. 1). The first tier aimed to identify as many individuals as possible with general psychological distress and prevent further assessment of individuals with no symptoms. The second tier differentiates between different types of symptoms, also functioning as a gateway to further assessment. Finally, the third tier indicates severity of symptoms. For this study, the Tier 3 scales were used as the reference standard, posing as proxies for symptom severity levels and identifying clinically relevant symptoms of specific disorders. In this study, all included scales (see below) have been answered separately. That is, redundant items (e.g. items 1–2 of the PHQ-9 equals PHQ-2) have been answered twice.

#### Symptom scales

Symptom scales have been selected based on sound psychometric properties, cross-cultural validity, and/ or previous use in refugee populations. Some of the scales used have been translated and validated by us, in previous projects [18, 34]. Others were translated for the present study (PCL-5 to Dari, ISI-7 to Farsi, and SGTI to Dari, Farsi, and Arabic for this study). We implemented a rigorous translation procedure to ensure the instruments’ semantic, conceptual, and cultural equivalence. The procedure followed Douglas and Craig’s collaborative iterative questionnaire translation [35]. The steps included professional translators first translating the symptom scales; then reviewing translations by bilingual project personnel. Our bilingual staff had a background in healthcare, were fluent in the target language and had high proficiency in English, Swedish, or both. Finally, an expert panel, decided on final amendments. After that, all material, including information, was discussed in focus groups consisting of individuals from the target group, led by bilingual staff and a research team member. We had further discussions in the expert panel regarding potential problems, if necessary. Some scales, translated in a previous project (i.e. the RHS-13, PHQ-9, and GAD-7, see below), also went through back translation and another discussion in the expert panel. However, by experience, we did not find that this step added to the quality of the translations.

**General emotional distress.** To screen for general psychological distress in Tier 1, we used the Refugee Health Screener, 13-item version, RHS-13 [36, 37]. The RHS was developed as a culturally sensitive brief first screener for refugees, screening for emotional distress aiming to detect individuals with symptoms of Depression, Anxiety and PTSD. The scale consists of 13 items scored from 0 to 4 on a Likert scale, yielding a maximum score of 52. A cut-off score of 11 or more has been established as a sensitive cutoff for identifying symptoms of Depression, Anxiety and PTSD [36], and recent studies have also confirmed this cutoff [34].

**Depressive symptoms.** We used the Patient health questionnaire 9, PHQ-9 [38], to assess the symptom severity of Depression in Tier 3. The PHQ-9 consists of nine items scored from 0 to 3, yielding a maximum sum of scores of 27. The scale has shown good psychometric properties, and four cut-offs have been established for identifying mild (≥ 5), moderate (≥ 10), moderately severe (≥ 15), and severe (≥ 20) symptoms of Depression [38]. For the present project, we used cutoff 15 and above, signifying the presence of major Depression, to estimate severe symptoms of Depression. The PHQ-9 has been used in clinical and research settings [39] and also in refugee populations [18, 34, 40].

The PHQ-2 consists of items 1 and 2 of the PHQ-9 (i.e. the two core criteria of Depression), and was used as the gateway for depressive symptoms in Tier 2. PHQ-2 has previously shown excellent operating characteristics for assessing major Depression with an optimal cut-off score of ≥3 (sensitivity 82.9%, specificity 90.0%, range 0–6 [41]).

**Anxiety symptoms.** Generalized Anxiety Disorder 7, GAD-7, was used to assess the symptom severity level of Anxiety in Tier 3 [42]. The scale consists of seven items (scored from 0 to 3, range 0–21) and has been used to identify general anxiety disorder using cut-off ≥10. GAD-7 has frequently been used to assess symptom severity in both clinical and research settings, also among refugees, using the cut-offs 5, 10, and 15 for mild, moderate, and severe symptoms, respectively [18, 34, 43].

The GAD-2 consists of items 1 and 2 of GAD-7, and the cut-off ≥3 has been used to screen for symptoms of anxiety disorders [44]. GAD-2 and PHQ-2 have been proposed as excellent first screeners, showing good coherence with the complete scales and clinical interviews. While initially developed to assess symptoms of generalized anxiety disorder, the GAD-7 and GAD-2 have also been found helpful for the initial identification of a range of anxiety disorders [45].

**Symptoms of PTSD.** We used the PTSD Checklist for DSM-5, PCL-5 [46], to measure the symptom severity of post-traumatic stress disorder (PTSD). The PCL-5 consists of 20 items with five labelled response alternatives, scored from 0 to 4 (range 0–80). Different cut-offs have been suggested for PCL-5, one more liberal cut-off at ≥28, an intermediate at 32, and a more conservative cut-off score at 38 [46–49]. For this study, we used these cut-offs to estimate mild, moderate and severe symptoms of post-traumatic stress in Tier 3.

To identify symptoms of PTSD in Tier 2, we compared two different gateways. The PCL Short Form, PCL-SF [49], a 4-item short form of the PCL-5, and the Single General Trauma Item, SGTI [50], indicating the occurrence of a potentially traumatic event. PCL-SF consists of items 3, 7, 13 and 15 from the PCL-5, scored from 0 to 4 (range 0–16), and it has shown excellent correspondence with the full PCL-5 scale. The SGTI has a “yes” or “no” alternative to indicate that the individual has experienced a potentially traumatic event.

**Symptoms of Insomnia.** The Insomnia Severity Index, ISI-7 [51], was used to measure the symptom severity of Insomnia. ISI-7 consists of seven items scored from 0 to 4 (range 0–28). The scale has three cut-offs: a sum of ≥8 indicates sub-threshold Insomnia, ≥ 15 indicates moderate Insomnia, and ≥ 22 indicates severe Insomnia. In this study, we have used these cut-offs to indicate mild, moderate, and severe symptoms of sleep insomnia. ISI-7 has been translated into several languages and used in various populations, including refugee populations [13, 52].

For the identification of symptoms in Tier 2, we tested two gateways. The first, a single item from the World Health Organization Quality of Life – brief version, (WHOQOL-BREF item 16, *How satisfied are you with your sleep?* [53]), and a similar item included in the ISI-7 (item 7 in the current version of ISI, *How worried/distressed are you about your current sleep problem?*). The item from WHOQOL was reversed and ranged from 1 to 5.

### Analytical process

We have investigated the occurrence of any essential differences between response language groups (Arabic [*n* = 393], Dari [*n* = 95], Farsi [*n* = 75], English [*n* = 59], and Swedish [*n* = 135]), regarding means, correlations, and internal consistency. Essential differences herein are psychometric problems with specific scales in certain languages that could affect the screening procedure.

The total sample size is well above any minimum for the analyses conducted. As indicated by previous studies, the prevalence of moderate symptoms for each disorder was estimated to be above 30%. Focusing on high sensitivity, we used an estimated prevalence of 20%, requiring a minimum of 100 participants to achieve 80% power (alfa set to 5%) for detecting differences between a screening test with 50% sensitivity (the null hypothesis) and a test sensitivity of 80% (the alternative hypothesis [54]). Considering that we investigated the prevalence of four different conditions, we set the minimum sample size to 400 participants.

Tier 1 aimed to identify as many individuals as possible with clinically relevant symptoms. Therefore, we used the previously established sensitive cut-off 11 for RHS-13, to investigate whether this cut-off efficiently identified many individuals with moderate symptoms of Depression, Anxiety, PTSD, and Insomnia. Screening accuracy, using RHS-13, was calculated for each proxy’s symptom severity level.

Tier 2 aimed to differentiate between symptoms using brief gateway instruments. We used a more exploratory approach in identifying gateways and optimal cut-offs of the gateways, as part of developing the procedure. We tested the relation between the gateway and the proxy, and analysed the overlap using Receiver Operating Characteristics (ROC) analysis. Youden’s *J* was used to indicate the optimal performance of the cut-offs identified. Developing a screening procedure, we prioritised the ability to identify occurrences of symptoms in the tradeoff between sensitivity and specificity. Thus, our criteria for identification of cut-offs were: 1. sensitivity above 80%, 2. specificity above 50%, and 3. higher sensitivity compared to specificity.

In order to evaluate the possible efficiency of using a tiered screening procedure, we demonstrate two different models. These models exemplify two potential uses for identifying moderate symptoms of Depression, Anxiety, PTSD, and Insomnia: one aiming to identify as many moderate symptoms as possible, and one aiming to make as many correct positive and negative assessments as possible. We have calculated accuracy, change in unnecessarily assessed individuals (false positives), and item burden for both models.

Individuals with incomplete data were excluded scale-wise. Due to a technical issue, only 718 individuals had responses on PCL-5 and 748 had responses on ISI-7. In total, 709 individuals had complete data, and only individuals with complete data were included when calculating the models. All analyses were conducted with IBM SPSS Statistics 27.

## Results

### Descriptive of the scales

The results showed no differences between language groups, regarding distribution, correlation, or internal consistency. Each scale exhibited very good internal consistency over all languages, with total Chronbach’s Alpha .91 for RHS-13 (*n* = 757), .91 for PHQ-9 (*n* = 757), .92 for GAD-7 (*n* = 757), .96 for PCL-5 (*n* = 718), and .86 for ISI-7 (*n* = 748). All scales were intercorrelated, *r* ranging from .64–.86 (all *p* < .001). Symptom burden was generally high (see Table 2), with mean scores close to, or above, moderate symptom levels on all scales. RHS-13 showed a mean of 22.27 (*SD* = 11.90, 95% CI [21.39, 23.15]), the mean for PHQ-9 was 12.92 (*SD* = 7.24, 95% CI [12.38, 13.45]), for GAD-7, 9.72 (*SD* = 6.05, 95% CI [9.27, 10.17]), for PCL-5, 35.57 (*SD* = 20.43, 95% CI [34.07, 37.08]), and finally, for ISI-7 it was 13.59 (*SD* = 7.07, 95% CI [13.07, 14.11]). Comorbidity was also very high, with as many as 433 individuals with moderate symptoms on two or more scales, of which 248 had moderate symptoms on all scales.

**Table 2:** Tier 1 analysis. Sensitivity and specificity from the Tier 1 screening (RHS-13, cutoff ≥ 11) using full scale screeners for mild, moderate, and severe symptoms of Depression (PHQ-9), Anxiety (GAD-7), PTSD (PCL-5), and Insomnia (ISI-7) as reference standard

Proxy	*N*_*pos*_	Sensitivity (%)	Specificity (%)	PPV (%)	NPV (%)	Effectiveness (%)
***PHQ-9*** (*N* = 757)						
Mild (cutoff 5)	631	87.6	77.8	95.2	55.7	86.0
Moderate (cutoff 10)	466	96.6	55.0	77.5	90.9	80.6
Severe (cutoff 15)	318	98.7	39.2	54.0	97.7	64.2
***GAD-7*** (*N* = 757)						
Mild (cutoff 5)	560	92.3	67.5	89.0	75.6	85.9
Moderate (cutoff 10)	364	98.9	43.8	62.0	97.7	70.3
Severe (cutoff 15)	177	99.4	30.2	30.3	99.4	46.4
***PCL-5*** (*N* = 718)						
Mild (cutoff 28)	448	97.3	49.6	76.2	91.8	79.4
Moderate (cutoff 32)	399	98.7	44.2	68.9	96.6	74.5
Severe (cutoff 38)	325	99.7	36.9	56.6	99.3	65.3
***ISI-7*** (*N* = 748)						
Mild (cutoff 7)	594	86.7	62.3	89.9	54.9	81.7
Moderate (cutoff 14)	372	94.9	41.5	61.6	89.1	68.0
Severe (cutoff 21)	107	99.1	27.1	18.5	99.4	37.4
***ANY*** (*N* = 709)						
Mild	648	85.3	82.0	98.0	34.5	85.1
Moderate	522	94.6	62.6	87.6	80.7	86.2
Severe	395	98.5	44.3	69.0	95.9	74.5

### Tier 1 analysis – identification of symptoms

Prevalence of emotional distress was indicated among 76.8% of all participants (581/757), showing scores of 11 and above on the RHS-13. This cut-off identified the majority of all participants that had any symptoms (85.3%, i.e. from mild and above, 553/648) on any of the four symptom scales (PHQ-9, GAD-7, PCL-5, and ISI-7), and 94.6% (494/522) of those with clinically relevant symptoms (i.e. from moderate symptoms and above; see Table 2 for sensitivity and specificity for all symptom levels of each scale).

### Tier 2 analysis – differentiation between symptoms

To optimise Tier 2, operating characteristics and performance for all cut-offs of each psychiatric disorder (Depression, Anxiety, PTSD, and Insomnia) identified within the predefined criteria (sensitivity ≥80%, specificity ≥50%, and sensitivity > specificity) were calculated (see Table 3 for a summary). Comparisons between the intercorrelations for all response languages showed no differences.

**Table 3:** Tier 2 analysis. Sensitivity and specificity of the Tier 2 gateways to each proxy screener for moderate symptoms of Depression (PHQ-9), Anxiety (GAD-7), PTSD (PCL-5), and Insomnia (ISI-7). The table shows all cutoffs within the predefined criteria (sensitivity ≥ 80%, specificity ≥ 50%, and sensitivity > specificity)

Proxy	AUC	Sensitivity (%)	Specificity (%)	PPV (%)	NPV (%)	Effectiveness (%)	Youden’s *J*
**PHQ-9** (*n* = 757)	.909						
*PHQ-2* Cutoff 2		97.0	50.5	75.8	91.3	79.1	.475
Cutoff 3		85.0	83.2	89.0	77.6	84.3	.681
**GAD-7** (*n* = 757)	.919						
*GAD-2* Cutoff 2		97.3	51.9	65.2	95.3	73.7	.492
Cutoff 3		86.0	85.8	84.8	86.9	85.9	.717
**PCL-5** (*n* = 718)	.946						
PCL5-SF Cutoff 4		97.7	55.5	73.3	95.2	79.0	.532
Cutoff 5		95.7	73.4	81.8	93.2	88.2	.691
Cutoff 6		89.7	83.1	86.9	86.6	92.5	.728
**ISI-7** (*n* = 748)	.928						
ISI7-i7 Cutoff 2		90.9	83.0	84.1	90.2	86.9	.738

#### Depressive symptoms

The gateway for PHQ-9 was PHQ-2. As could be expected, the scales showed a strong positive correlation, *r*(755) = .82, *p* < .001, and excellent operating characteristics, with area under the curve (AUC) = .909 (95% CI [.888, .930]). Cut-off ≥2 showed higher sensitivity (97.0%) compared to cut-off 3 (85.0%). However, cut-off 3 showed a better overall performance (Youden’s *J =* .682) compared to cut-off 2 (*J* = .475), due to a much higher specificity at cut-off 3 (83.2 and 50.5% for cut-off 3 and 2, respectively).

#### Anxiety symptoms

The gateway for GAD-7 was GAD-2. As with the PHQ, the GAD scales were also strongly positively correlated, *r*(755) = .84, *p* < .001, showing excellent operating characteristics, AUC .919 (95% CI [.900, .938]). Cutoff ≥2 showed higher sensitivity (97.3%) compared to cutoff ≥3 (86.0%), with a better overall performance at cutoff ≥3 (*J =* .717) compared to cutoff ≥2 (*J* = .492), due to the higher specificity for cutoff ≥3 (85.8 and 51.9% for cutoff 3 and 2, respectively).

#### Symptoms of PTSD

The gateways tested for PCL-5 were PCL-SF and SGTI. PCL-SF outperformed SGTI in predicting symptoms on PCL-5. PCL-SF showed a strong positive correlation with PCL-5, *r*(716) = .84, *p* < .001, and AUC was .946 (95% CI [.930, .961]). Not falling below 50% specificity, the highest sensitivity reached 97.7% (specificity 55.5%, *J* = .532) at cut-off ≥4. However, already at cut-off ≥5 specificity improved considerably (73.4%), maintaining a high sensitivity (95.7%, *J* = .691). The highest Youden statistics, keeping sensitivity higher than specificity was reached at cut-off ≥6 (*J* = .728, sensitivity 89.7%, specificity 83.1%). SGTI showed a weak positive correlation to PCL-5, *r*(716) = .23, *p* < .001, with sensitivity 84.2% and specificity 37.6% for its sole cut-off. The PCL-SF was, therefore, determined to be the preferred gateway.

#### Symptoms of insomnia

Also for ISI-7 two gateways were tested, item 16 from the WHOQOL-BREF and item 7 from ISI-7 (ISI7-i7). ISI7-i7 showed the highest communality (*h*^*2*^ = .743) of all items included in the full scale, and had a factor loading of .862. ISI7-i7 performed better than item 16 from WHOQOL-BREF, with AUC = .928, (95% CI .910, .946]; compared to AUC = .861 for item 16). Sensitivity and specificity for ISI7-i7, cut-off ≥2, were 90.9 and 83.0%, respectively (*J* = .738) as compared to sensitivity 77.2% and specificity 81.6% for item 16 at cut-off ≥3). The ISI7-i7 was therefore kept as the gateway to ISI-7 with one single cut-off.

### Evaluation of screening efficiency

In order to test the screening efficiency of a full tiered procedure, a model intended to capture as many individuals with clinically relevant symptoms as possible (i.e. high sensitivity) was compared to a model aiming at precision (i.e. as many both positively and negatively correctly identified individuals as possible). The differences between the models are implemented by using different cut-offs in the Tier 2 gateways. For demonstrating a model with high sensitivity, we used the cut-offs with the highest sensitivity for the gateways, as identified in the Tier 2 analysis. However, we chose cut-off 5 for the PCL-SF since it increased specificity by almost 20% from (55.5 to 73.4%), only reducing sensitivity by 2% (from 97.7 to 95.7%). Aiming for optimal scale performance in the precision model, we used the cut-offs rendering the highest Youden statistics. Symptoms of Insomnia were assessed similarly in both models due to only one cut-off falling within the criteria we used. Thus, for the sensitivity model, cut-off 2 for PHQ-D, PHQ-A, and ISI-i7, and 5 for PCL-SF, were used, whereas the precision model used cut-off 3 for PHQ-D and PHQ-A, and cut-off 6 for PCL-SF. The models were calculated on data from all individuals that had completed all scales (*n* = 709). The prevalence of moderate symptoms was high in all symptom categories and comorbid symptom was common. Because of that, many individuals were rejected for some of the next tier scales while eligible for others. We, therefore, refer to individual assessments of each scale as individual scale response events (ISRE, i.e. each time an individual responded to one unique scale).

#### Screening efficiency: tier 1

In Tier 1, 79.5% of all participants (564/709) indicated symptoms of emotional distress. As stated previously, 94.6% of the 522 individuals displaying moderate symptoms and above on any of the proxies were correctly identified by the RHS-13 (i.e. true positive), thus missing a total of 28 individuals (i.e. false negative), of which 6 indicated severe symptoms. Of all individuals indicating no symptoms on any proxy, 62.6% (117/187) were correctly rejected (true negative), rendering a total of 70 individuals falsely positively forwarded (i.e. false positive). Utilising Tier 1 reduced the item burden by 69.8% (13 items, compared to 43 items for all full-length scales) for all non-symptomatic individuals. A total of 564, (494 true positives + 70 false positives) individuals were forwarded to Tier 2, rendering a total burden of 24,252 items (564 individuals × 43 items, using full-scale references). The total effectiveness (i.e. the percentage of correct positive and negative assessments) of Tier 1 alone was 86.2%.

#### Screening efficiency: model testing

Tier 2 of the sensitivity model could identify 99.6% of the participants with moderate symptoms on any scale, forwarded from Tier 1. In contrast, the precision model identified 96.2% (see Fig. 1 for a comprehensive overview of each specific symptom). Of the 70 false positives in Tier 2, 9 individuals could be correctly rejected in the sensitivity model, and 37 in the precision model. However, 19 individuals with moderate symptoms on any scale were also falsely rejected in this model (3 with severe symptoms). In contrast, the sensitivity model rendered only two more false negatives (1 with severe symptoms).

Utilising Tier 2 would reduce the total item burden by 48.8% for all non-symptomatic individuals (13 items from Tier 1 + 9 items from Tier 2, compared to 43 items for all full-length scales). In this sample, using the Tier 2 gateways could also reduce the total number of false positive assessments (i.e. unnecessarily answered scales) forwarded from Tier 1 with 51.2% in the sensitivity model (from 728 to 355 false positive ISREs) and 76.2% in the precision model (to 173 false positive ISREs). Moreover, the total number of correctly rejected ISREs in Tier 2 was much higher in the precision model (549 true negative ISREs), compared to the sensitivity model (373 true negative ISREs). Utilising Tier 2, thus rendered an item burden reduction of 15.8% (to 20,416 items) and 24.6% (to 18,276 items) for the sensitivity and precision models, respectively.

**Fig. 2:**
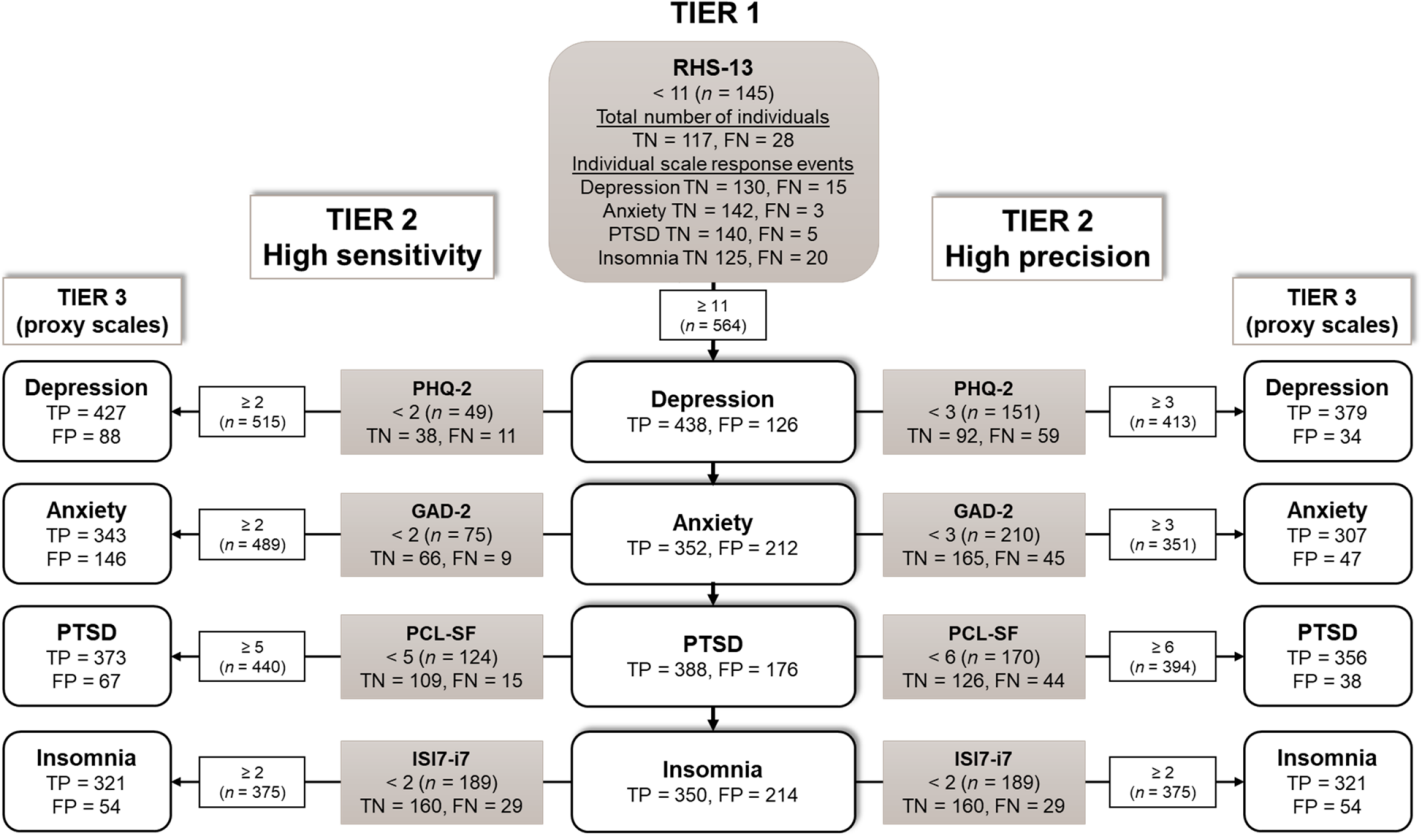
Model testing A comparison between two different tiered screening processes, one aims for high sensitivity (left) and the other for high precision (right). The figure shows all scale response events from all individuals screened (N = 709). Scales from Tier 1 and Tier 2 were tested against each symptom category’s proxy screener (Tier 3) for moderate symptoms of Depression (PHQ-9), Anxiety (GAD-7), PTSD (PCL-5), and Insomnia (ISI-7). For a better understanding of the screening process, all negative assessments (in gray) are presented when rejected as TN (true negative) and FN (false negative). In contrast, the positive assessments (in white) are forwarded as TP (true positive) and FP (false positive)

The total effectiveness was calculated with true and false positives based on the outcome of Tier 2, and the sum of true and false negatives from Tier 1 and Tier 2. The precision model showed better overall effectiveness (86.1%) than the sensitivity model (83.7%). See Fig. 2 for a full overview of both models.

## Discussion

The purpose of the present study was to develop and evaluate an online procedure adapted for refugee populations, to screen for clinically relevant symptoms of Depression, Anxiety, PTSD, and Insomnia. The results show that the tiered procedure i-TAP, herein tested in two models (a sensitivity model and a precision model), could be used as a screening tool to efficiently identify and differentiate between symptoms of several psychiatric disorders among refugees. We optimized screening efficiency by identifying optimal gateways and cut-offs in the i-TAP.

In Tier 1, using an initial general screener, a large proportion of the individuals with no symptoms could be correctly prevented from further screening, reducing their item burden by nearly 70%, while forwarding almost 95% of people with clinically relevant symptoms for further assessment. As expected, many false positives from Tier 1 moved to Tier 2 due to using a highly sensitive first screener (i.e. the RHS-13). However, using the gateways in Tier 2 helped specify symptoms and could further reduce the number of unnecessary individual assessments (false positive ISREs), and thus, the total item burden. Accuracy remained high over both tiers for both models, with no reduction over Tiers in the precision model (86% after Tier 1 and 2 both). Even if the sensitivity model left fewer participants with a false negative assessment, the total number of individuals with any moderate symptoms, or above, not captured by any of the models, was generally very low, with a slight difference between models. As demonstrated in both models, this tiered procedure could efficiently reduce unnecessarily posed and answered questions while maintaining high accuracy.

Reducing the item burden reduces individual efforts, increasing the probability of initiating and finalising a screening. However, the reduced item burden rendered by the i-TAP will foremost apply to individuals with no or low levels of psychological symptoms. For example, non-symptomatic individuals correctly assessed in Tier 1 would get a reduced item burden by almost 70%. In contrast, an individual with, for example, symptoms of Depression would get a reduced item burden by about 30%, compared to using full-length scales. In this sample, comorbid symptoms were highly prevalent, and about a third of the respondents had moderate symptoms or above on all scales (*n* = 248). For individuals with symptoms within several categories, the reduction of items is lower, and there is even an increased item burden for those with symptoms within all categories. Considering the high symptom levels in this sample and the relation between the prevalence of symptoms and item burden, it is highly plausible that the item reduction would be much more significant in the entire refugee population.

Despite the increase of items for individuals with high comorbidity, we argue that adding the RHS-13, a tool developed for and adapted to refugees, contributes to the screening procedure. It comprises culturally sensitive descriptions of symptoms designed specifically for this heterogeneous population, also including somatic symptoms [36]. The RHS was developed from The New Mexico Refugee Health Symptom Checklist-121 [55], which includes a broad range of physical and mental symptoms experienced by refugees. Although beyond the scope of this study, it is crucial to also assess somatic distress among refugees since somatic symptoms are common among individuals with traumatic experiences and are often what is first presented when individuals with mental health problems contact healthcare services [56]. The RHS-13 works well as a first step to approach mental health issues and thus can serve as a bridge to touch on more specific symptoms. Therefore, it poses a suitable entry in a tiered mental health screening, as an effective general screener in Tier 1 and a gentle introduction for those who will continue to the gateways and complete scales in Tier 2 and 3.

The high prevalence and comorbidity rate, shown in this study and confirmed in previous research [6, 13], points to the importance of screening refugees for multiple mental health problems. With a model like the i-TAP this is easily achieved, as it screens for several psychiatric disorders simultaneously. Instead of deciding which disorders to screen for in each case, with the risk of missing essential symptoms, a tiered procedure can be administered. This, together with the increased screening efficiency, could lower the threshold for service providers to suggest a mental health screening, resulting in more individuals being screened, identified, and eventually treated for mental health problems. When used in a clinical setting, it could also serve as a way to initiate difficult conversations regarding mental health issues. Findings from our research (manuscript in preparation), indicate that the RHS could be an excellent gate opener in these regards. We have every reason to believe the full i-TAP could serve the same function.

Furthermore, the i-TAP could facilitate decision-making in psychiatric and primary care settings, by guiding individuals to suitable online programs, identifying individuals in the most acute need of support and intervention, and suggesting potentially eligible participants for a trial. In other settings, such as schools or housing facilities, the i-TAP may help identify a need for healthcare support and motivate refugees to seek help when needed. As Batterham et al. [32] argued, a tiered screening toll might be particularly useful in service settings with time and resource constraints on assessing mental health statuses, such as primary care and schools.

From a user point of view, the opportunity to answer questions about mental health in one’s primary language, at one’s own pace, and at any time or place makes the i-TAP a potential bridge over some of the practical and economic barriers to mental health services reported by refugees [26, 27]. In summary, the i-TAP could be helpful in several settings. It poses a manageable, feasible, and affordable alternative to formal help-seeking and, therefore, could facilitate access to and delivery of mental health services for refugees.

As previously stated, the i-TAP can be adapted to fit a specific project or clinical practice’s needs, requirements, and resources. However, a tiered screening might not be a suitable option in some settings. For example, in a clinical or research settings where complete data on all measures is a requirement. It is also of great importance to stress that the i-TAP is a screening procedure, and it cannot replace a clinical diagnostic assessment. The function of the i-TAP is to constitute the first of several assessment steps. Furthermore, screening should always be followed by action plans and interventions.

The analyses led us to exclude two potential gateway items from the i-TAP, the sleep quality question from WHOOQOL-BREF and the Single General Trauma Item (SGTI), as they were outperformed by item 7 from Insomnia Severity Index (ISI-7) for Insomnia, and PCL-SF (for PTSD) respectively. Regarding the latter, this means that symptoms of trauma predicted more symptoms of trauma better than having experienced trauma, which is reasonable considering that the majority of those, who have experienced trauma, refugees included, do not develop PTSD [4, 57]. There is also a potential problem with the item itself, as reliability is affected by participants’ interpretation and labelling of trauma. On the other hand, trauma experience is necessary for PTSD diagnosis (criterion A in DSM-5), and a known risk factor for mental health problems [5, 12], motivating inquiry about trauma. However, our results show that it could be different. A recommendation for future studies could be to use the Refugee Trauma History Checklist [50]. However, since the i-TAP aims to screen for symptoms, the question about trauma experience could also wait until a further assessment is warranted.

## Limitations

The convenience sample and uncontrolled sampling method of this study is a limitation. In the aim, it is stated that we are developing a tool adapted for refugees. However, participants self-identified as refugees through participating (i.e. we explicitly recruited refugees) and answering control questions about nationality and if they had fled from their home country. Moreover, about 17% completed the survey in Swedish. We asked people to respond in the language that they felt most comfortable with, from a choice of five languages (Arabic, Dari, Farsi, English, and Swedish). Our experience is that many illiterate people cannot read or write in the first language but have learnt to read and write in the host language and in English, why we kept those versions. We should, however, have controlled for language proficiency. Furthermore, about half of the participants had no, or temporary residence permits. These circumstances are related to mental distress [18, 58], since they often imply loneliness, discrimination and language problems [59]. All of which may have affected the outcome of the screening procedure. However, targeting asylum housings in recruitment was intentional, as we wanted to reach groups needing adapted material. Despite these limitations, the sample is representative regarding the distribution of age, sex, country of origin, and region of residency in Sweden for the significant refugee populations residing in Sweden at the time of data collection [33]. Furthermore, the high prevalence of symptoms and comorbidity found is supported by previous studies using similar scales [18, 34], and we, therefore, believe that the psychometric evaluation is sufficient. However, it is essential to note that self-rating instruments tend to render a higher prevalence of symptoms than clinical interviews [9], which is related to another limitation. As of yet, the presented results regard the two first tiers of our model, validated with the full-length scales in Tier 3, self-rated and strongly related to the Tier 2 gateways, as reference standards. The full-length scales utilized have shown excellent psychometric standards when used in humanitarian settings and refugee populations [13, 34, 40, 60]. Even so, the results should be interpreted with some caution. For explanatory reasons, the project’s end goal is to validate the complete procedure including the third tier, assessing symptom severity, with a clinical interview. Finally, the selection of psychiatric diagnoses and scales limits the scope of the tired screening to conventional symptoms of Depression, Anxiety and PTSD. Future research and clinical practice may consider including other psychiatric symptoms (e.g. symptoms of complex PTSD) and a broader assessment of somatic symptoms.

## Conclusions

Based on the findings in this study, an online tiered screening procedure, like the i-TAP, could be utilised with good accuracy and efficiency to identify clinically relevant symptoms of multiple psychiatric disorders in refugee populations. Its adaptations, multi-symptom approach and potential to reduce the overall item burden could benefit both service users and providers, facilitating decision making and increasing access to mental health services in various settings.

## Data Availability

The datasets generated and analyzed during the current study are not publicly available as we have not completed writing on these data. Data are available from the corresponding author on reasonable request and will be made publicly available at completion.
